# Supplementary material for: Impact of Leptin on the Expression Profile of Macrophages during Mechanical Strain In Vitro
Source: Int J Mol Sci. 2022 Sep 14;23(18):10727. doi: 10.3390/ijms231810727 (PMC9503708; doi:10.3390/ijms231810727)
Supplement: Supplementary file 1 [file ijms-23-10727-s001.zip › ijms-1803970-supplementary.pdf]

International Journal of
Molecular Sciences

MDPI

Article

Impact of Leptin on Macrophages during Mechanical Strain

1. Uncropped Westernblots

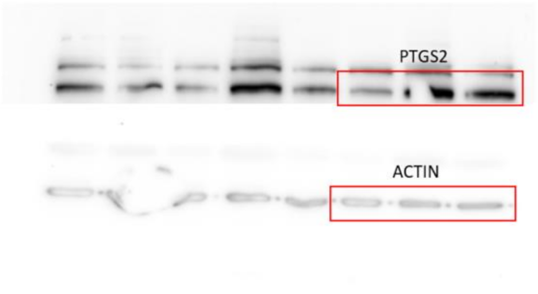

Figure S1. Uncropped Western Blot for Fig. 4h.

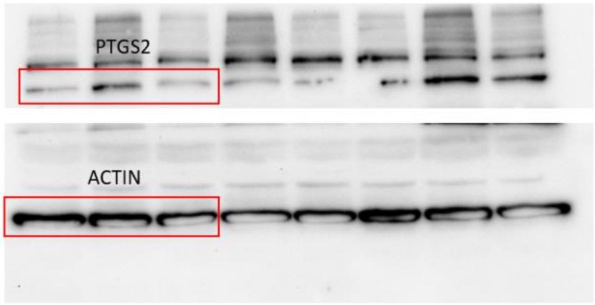

Figure S2. Uncropped Western Blot for Fig. 5h.

2 Cell Numbers and LDH Release after Different Conditions

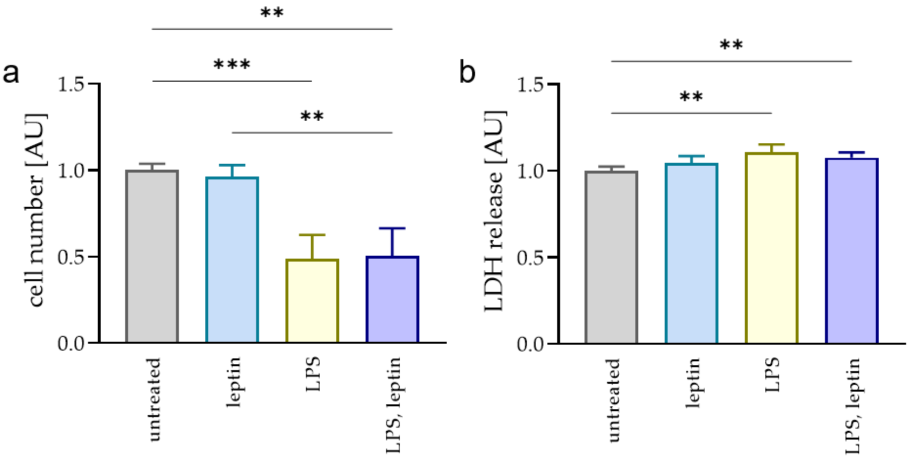

Figure S3. Impact of leptin, *Porphyromonas gingivals* LPS and a combination of both on cell number (a) and lactatdehydrogenase (LDH) release. n n=6; Statistics: Welch-corrected ANOVA with Dunnett's T3 multiple comparisons test; ** $P < 0.01$, *** $P < 0.001$.

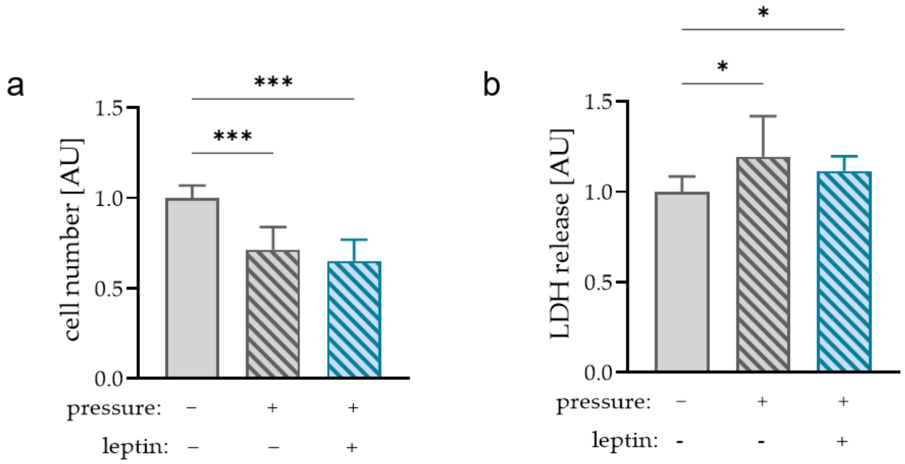

Figure S4. Impact of leptin in combination with compressive strain on cell number (a) and lactatdehydrogenase (LDH) release. n≥9; Statistics: Welch-corrected ANOVA with Dunnett's T3 multiple comparisons test; * $P < 0.05$, *** $P < 0.001$.

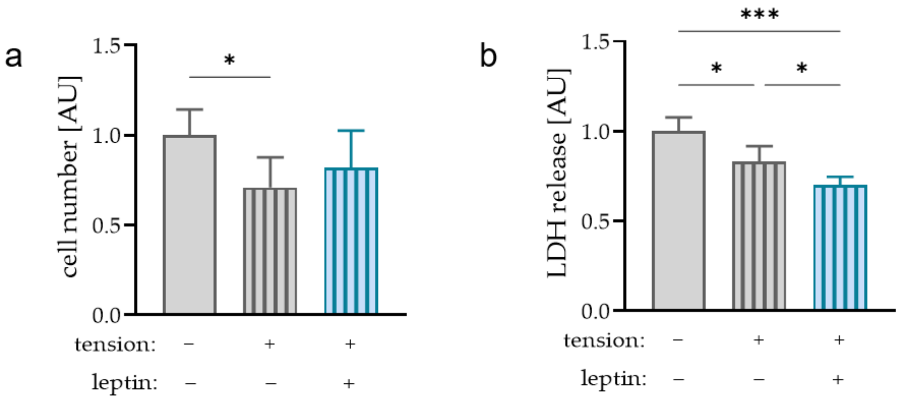

Figure S5. Impact of leptin in combination with tensile strain on cell number (a) and lactatdehydrogenase (LDH) release. n=6; Statistics: Welch-corrected ANOVA with Dunnett's T3 multiple comparisons test; * $P < 0.05$, *** $P < 0.001$.
